# The Effect of Asymptomatic and/or Treated Brain Metastases on Efficacy of Immune Checkpoint Inhibitors in Metastatic Non–Small Cell Lung Cancer: A Meta-Analysis

**DOI:** 10.3389/fonc.2021.702924

**Published:** 2021-06-25

**Authors:** Sihan Li, Hongwei Zhang, Tingting Liu, Jun Chen, Jun Dang

**Affiliations:** ^1^ Department of Radiation Oncology, The First Affiliated Hospital of China Medical University, Shenyang, China; ^2^ Department of Radiation Oncology, Anshan Cancer Hospital, Anshan, China; ^3^ Department of Radiation Oncology, Shenyang Chest Hospital, Shenyang, China

**Keywords:** immune checkpoint inhibitors, chemotherapy, non-small cell lung cancer, brain metastases, meta-analysis

## Abstract

**Background:**

To assess the effect of asymptomatic and/or treated brain metastases (BMs) on the efficacy of immune checkpoint inhibitors (ICIs) in metastatic non-small cell lung cancer (NSCLC).

**Patients and Methods:**

PubMed, Embase, Cochrane Library, Web of Science, and recent meetings were searched for randomized controlled trials (RCTs). The primary outcomes of interest were overall survival (OS) and progression-free survival (PFS).

**Results:**

Seventeen articles reporting 15 RCTs with 10,358 patients (1,199 with and 9,159 without BMs) were eligible. ICIs were associated with longer OS and PFS than those in chemotherapy either in patients with (hazard ratio [HR], 0.65; 95% confidence interval [CI], 0.51–0.82 and HR, 0.60; 95% CI, 0.45–0.79) or without BMs (HR, 0.74; 95% CI, 0.70–0.78 and HR, 0.70; 95% CI, 0.57–0.86); no significant difference in the pooled HRs for OS (P_interaction_ = 0.29) and PFS (P_interaction_ = 0.37) was observed between the two patient populations. Subgroup analyses revealed that either ICI monotherapy or combination therapy significantly improved OS and PFS compared with those in chemotherapy both for patients with and without BMs. Superior OS benefit from ICI combination therapy than that in monotherapy was observed in patients with BMs (HR, 0.49 *vs.* 0.81, P_interaction_ = 0.005) but not in patients without BMs (HR, 0.71 *vs.* 0.76, P_interaction_ = 0.27).

**Conclusion:**

There was no compelling statistical evidence that the efficacy of ICIs in metastatic NSCLC was modified by the presence of asymptomatic and/or treated BMs. Patients with BMs were likely to obtain more OS benefit from ICI combination therapy than that from monotherapy.

## Introduction

Brain metastases (BMs) are a common complication of advanced lung cancer with poor prognosis, occurring in 20% to 40% of patients with non-small cell lung cancer (NSCLC) (1). Currently, tyrosine kinase inhibitors (TKIs), especially third-generation TKIs, such as osimertinib and alectinib, have been recommended for epidermal growth factor receptor (EGFR) or anaplastic lymphoma kinase (ALK) mutations in NSCLC patients with BMs (2). However, for patients without these genetic aberrations, there are few satisfactory systemic treatment options. Recently, immune checkpoint inhibitors (ICIs) have changed the therapeutic landscape of metastatic NSCLC patients lacking EGFR or ALK alteration. However, the majority of ICIs trials systematically excluded patients with untreated/unstable BMs. Some recent RCTs (3–19) have included a small number of patients with asymptomatic and/or treated BMs but with inconsistent results. In CheckMate-057 (9), -078 (10), and a pooled analysis of KEYNOTE-010 and -024 and -042 trials (20), patients with baseline asymptomatic or treated BMs had similar OS with ICIs or chemotherapy (CT). Conversely, CheckMate-227 (11, 12), -9LA (13), and a pooled analysis of KEYNOTE-021 and -189 and -407 trials (21) showed that ICIs significantly improved survival compared with that in CT.

To date, no randomized-controlled trial (RCT) has specifically addressed the role of ICIs in NSCLC patients with BMs. Whether the presence of asymptomatic and/or treated BMs can affect the efficacy of ICIs remains uncertain. In light of this important issue, we conducted a meta-analysis to assess the efficacy of ICIs relative to CT in NSCLC patients with asymptomatic and/or treated BMs. In addition, differences in survival benefit from ICIs between patients with and without asymptomatic and/or treated BMs were also evaluated.

## Materials And Methods

### Literature Search Strategy

This meta-analysis was conducted in accordance with the Preferred Reporting Items for Systematic Reviews and Meta-analysis (PRISMA) criteria (22) (**Supplementary File**, **Table S1**). A systematic literature search of PubMed, Embase, Cochrane Library, and Web of Science up to November 10, 2020, was performed by two authors (LD and JQ) independently. Abstracts of recent international scientific meetings, including the American Society of Clinical Oncology (ASCO), European Society for Medical Oncology (ESMO), and World Conference on Lung Cancer (WCLC), were also inspected. The reference lists of relevant studies were checked for additional articles. The detailed search strategy is shown in **Supplementary File**, **Table S2**.

### Inclusion and Exclusion Criteria

Studies were included if they met the following criteria: (1) phase II and III trials in metastatic NSCLC; (2) compared ICIs (alone or in combination with other agents) with CT; (3) data regarding patients with and without BMs could be retrieved, respectively; (4) reported overall survival (OS) or progression-free survival (PFS) data in each arm; and (5) published in English. Retrospective studies were not considered eligible. If studies had multiple publications, the most recent one was used. Conference abstracts could be included in the meta-analysis if they reported OS and/or PFS data according to patients’ BMs status.

### Data Extraction

Two authors (SL and HZ) independently extracted the following information from each included trial: trial name/first author, design, region, number of patients with and without BMs, interventions, hazard ratios (HRs), and their 95% confidence intervals (CIs) of OS and PFS.

### Quality Assessment

The risk of bias of individual studies was assessed by two authors (SL and HZ) independently, using the Cochrane Risk of Bias Tool (23), which consists of the following domains: sequence generation, allocation concealment, blinding, incomplete data, and selective reporting. The studies were finally classified as low (all domains indicated as low risk), high (one or more domains indicated as high risk), and unclear risk of bias (more than three domains indicated as unclear risk).

### Statistical Analysis

Statistical analysis was performed using the software Review Manager 5.3 (Cochrane Collaboration, Oxford, UK). The primary outcomes of interest were OS and PFS. HRs and their 95% CIs were used as summary statistics. A statistical test for heterogeneity was conducted using the Chi-square (χ^2^) and I-square (I^2^) test with significance set at P < 0.10 and/or I^2^ > 50%. If significant heterogeneity existed, a random-effects analysis model was used; otherwise, a fixed-effects model was used. In addition, we performed subgroup analyses according to ICI monotherapy, ICI combination therapy, first-line treatment with ICIs, and subsequent-line treatment with ICIs. The differences in the effect of ICIs were assessed using the χ^2^ test and expressed as P for interaction. The stability of the pooled results was evaluated by a sensitivity analysis in which the data of an individual study were removed each time. The funnel plot, Begg’s test (24), and Egger’s linear regression test (25) were performed to investigate any potential publication bias. *P-*values < 0.05 were generally considered statistically significant. However, for multiple interaction tests in subgroup analyses, a *P*-value of 0.05÷K (K, number of subgroups) was used as the threshold for significance in light of the correction for multiplicity (26).

## Results

### Literature Search and Study Selection

**Figure 1:**
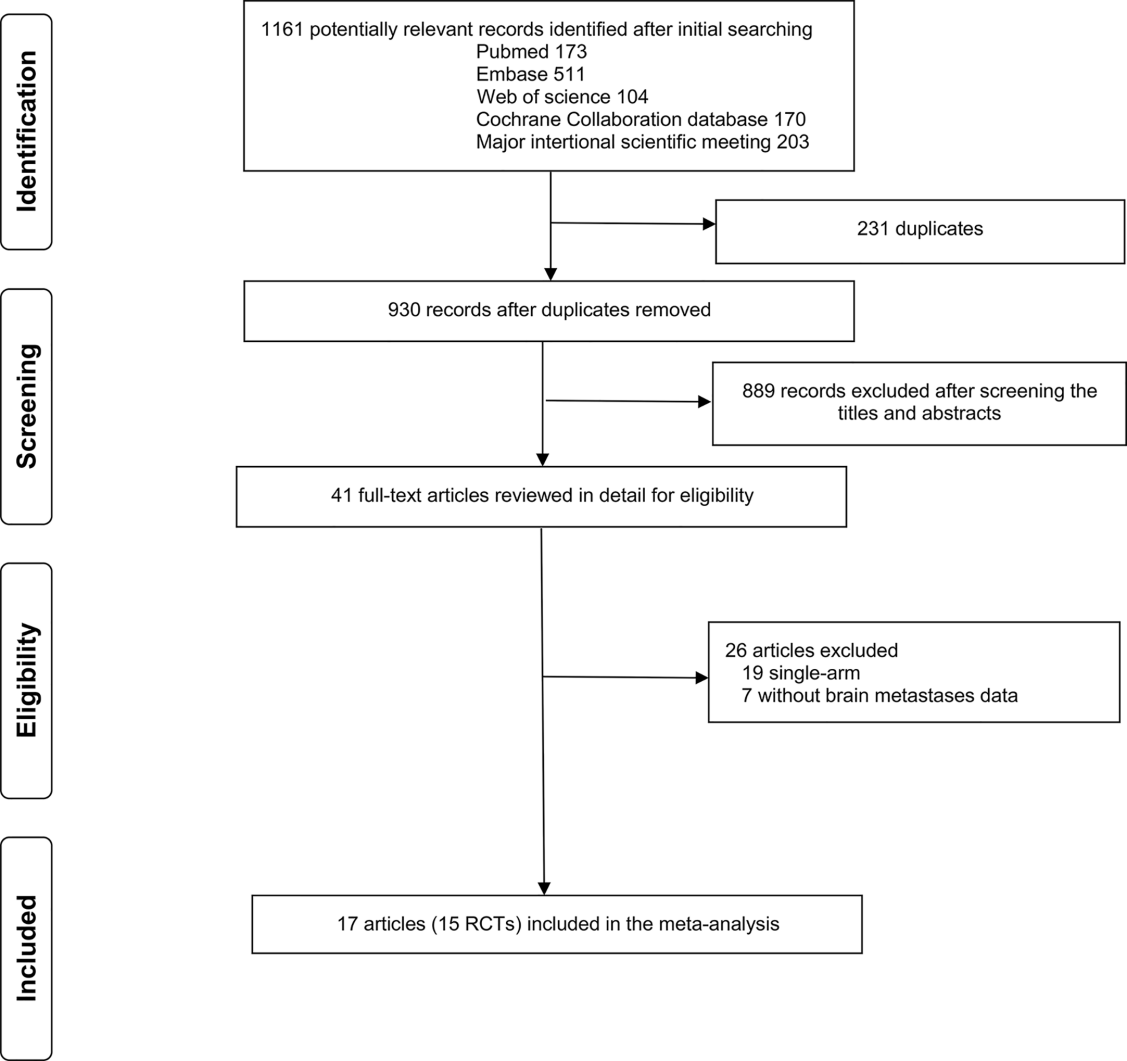
Literature search and selection. RCTs, randomized control trials.

A total of 1,161 studies were identified from the initial literature search (n = 173 for PubMed, n = 511 for Embase, n = 104 for Web of science, n = 170 for Cochrane Library, and n = 203 for meetings), and 41 potentially eligible reports were retrieved for detailed review (**Figure 1**). The relevant references were also reviewed for missed studies. Finally, 17 eligible articles (3–19) reporting 15 RCTs (14 phase 3 and 1 phase 2 trials) with 10,358 patients (1,199 with and 9,159 without asymptomatic/treated BMs) were included in the meta-analysis. Most of the RCTs (3, 5–9, 11–18) stated clearly that patients with meningeal metastasis were excluded, whereas the other three trials (4, 10, 19) did not provide information for whether patients with meningeal metastasis were excluded. The clinical and demographic characteristics of included studies are shown in **Table 1** and **Supplementary File**: **Table S3**. Twelve studies provided OS data, and 13 studies reported PFS data. Given that two studies (20, 21) provided pooled data of KEYNOTE-010 (3), -024 (4), and -042 (5) trials, and KEYNOTE-021 (6), -189 (7), and -407 (8) trials, respectively, the pooled data were used instead of data from the individual trials in this meta-analysis. The median sample sizes of BMs and non-BMs arms were 72 participants (range: 15–152) and 514 participants (range: 277–1204), respectively.

**Table 1:** Clinical characteristics of included trials.

Trial/Year	Phase	Treatment line	Primary endpoint	Median follow-up (months)	Treatment	Size (with/without BMs)	ICIs class	With BMs		Without BMs	
								OS	PFS	OS	PFS
								HR (95% CI)	HR (95% CI)	HR (95% CI)	HR (95% CI)
Keynote-010/2016 (3)	3	≥2	OS, PFS	13·1	Pembrolizumab	104/586	Anti-PD-1	0.83 (0.62–1.10)	0.96 (0.73–1.25)	0.78 (0.71–0.85)	0.91 (0.84–0.99)
					Doctaxel	48/295					
Keynote-024/2016 (4)	3	1	PFS	11.2	Pembrolizumab	18/136	Anti-PD-1	0.83 (0.62–1.10)	0.96 (0.73–1.25)	0.78 (0.71–0.85)	0.91 (0.84–0.99)
					PP/GP/PC	10/141					
Keynote-042/2019 (5)	3	1	OS	12.8	Pembrolizumab	35/602	Anti-PD-1	0.83 (0.62–1.10)	0.96 (0.73–1.25)	0.78 (0.71–0.85)	0.91 (0.84–0.99)
					PC/PP	35/602					
Keynote-021/2016 (6)	2	1	ORR	10.6	Pembrolizumab+PP	9/51	Anti-PD-1	0.48 (0.32–0.70)	0.44 (0.31–0.62)	0.63 (0.53–0.75)	0.55 (0.48–0.63)
					PP	6/57					
Keynote-189/2018 (7)	3	1	OS, PFS	10.5	Pembrolizumab+PP	73/337	Anti-PD-1	0.48 (0.32–0.70)	0.44 (0.31–0.62)	0.63 (0.53–0.75)	0.55 (0.48–0.63)
					PP	35/171					
Keynote-407/2018 (8)	3	1	OS, PFS	7.8	Pembrolizumab+PC/CnP	20/258	Anti-PD-1	0.48 (0.32–0.70)	0.44 (0.31–0.62)	0.63 (0.53–0.75)	0.55 (0.48–0.63)
					PC/CnP	24/257					
CheckMate-057/2015 (9)	3	≥2	OS	13.2	Nivolumab	34/258	Anti-PD-1	1.04 (0.62–1.76)	0.80 (0.47–1.36)	0.71 (0.58–0.88)	0.92 (0.76–1.12)
					Doctaxel	34/256					
CheckMate-078/2019 (10)	3	≥2	OS	8.8	Nivolumab	45/293	Anti-PD-1	0.82 (0.42–1.60)	0.62 (0.35–1.10)	0.70 (0.53–0.92)	0.79 (0.62–1.00)
					Doctaxel	27/139					
CheckMate-227/2019 (11, 12)	3	1	OS	28.3	Nivolumab+Ipilimumab	64/519	Anti-PD-1+Anti-CTLA-4	0.64 (0.42–0.98)	NR	0.75 (0.64–0.88)	NR
					Platinum-based	52/532					
CheckMate-9LA/2020 (13)	3	1	OS	8·1	Nivolumab+Ipilimumab+CT	65/296	Anti-PD-1+Anti-CTLA-4	0.38 (0.24–0.61)	NR	0.75 (0.61–0.92)	NR
					CT	57/301					
OAK/2019 (14, 15)	3	≥2	OS	21	Atezolizumab	61/364	Anti-PD-L1	0.74 (0.49–1.13)	0.38 (0.16–0.91)	0.74(0.63-0.88)	0.99 (0.50–1.97)
					Doc	62/363					
SHR-1210-303/2019 (16)	3	1	PFS	11.9	Camrelizumab+PC	10/194	Anti-PD-1	NR	0.14 (0.01–0.88)	NR	0.61 (0.46–0.81)
					PC	6/201					
ORIENT-11/2020 (17)	3	1	PFS	8.9	Sintilimab+PP	36/230	Anti-PD-1	NR	0.58 (0.28–1.18)	NR	0.47 (0.34–0.64)
					PP	22/109					
EMPOWER-Lung1/2020 (18)	3	1	PFS、OS	10.8	Cemiplimab	44/312	Anti-PD-1	0.44 (0.19–1.07)	0.49 (0.27–0.90)	0.71(0.54-0.92)	0.62 (0.51–0.76)
					CT	39/315					
Lee/2020 (19)	3	1	PFS	7.4	Nivolumab+PC+Bev	36/239	Anti-PD-1	NR	0.65 (0.36–1.18)	NR	NR
					PC+Bev	41/234					

OS, overall survival; PFS, progression-free survival; ORR, objective response rate; HR, hazard ratio; 95%CI, confidence interval; ICIs, immune checkpoint inhibitors; BMs, brain metastases; CT, chemotherapy; PP, pemetrexed-cisplatin/carboplatin; PC, paclitaxel-carboplatin; CnP, paclitaxel-nanoparticle albumin-bound-carboplatin; GP, gemcitabine-cisplatin; Bev, bevacizumab; NR, not reported.

### Assessment of Included Studies and Publication Bias

The risk of bias in included RCTs is summarized in **Supplementary File**, **Figure S1**. Only one trial (19) was judged as having an unclear risk of bias, as it had more than three domains for indicating them an unclear risk. The remaining trials were rated with a low risk of bias. The Begg’s and Egger’s test results indicated no publication bias in OS (P = 0.71 and P = 0.57) and PFS (P = 0.12 and P = 0.99). The funnel plot is shown in **Supplementary File**, **Figure S2**.

### Effect of ICIs on OS and PFS in Patients With and Without BMs

**Figure 2:**
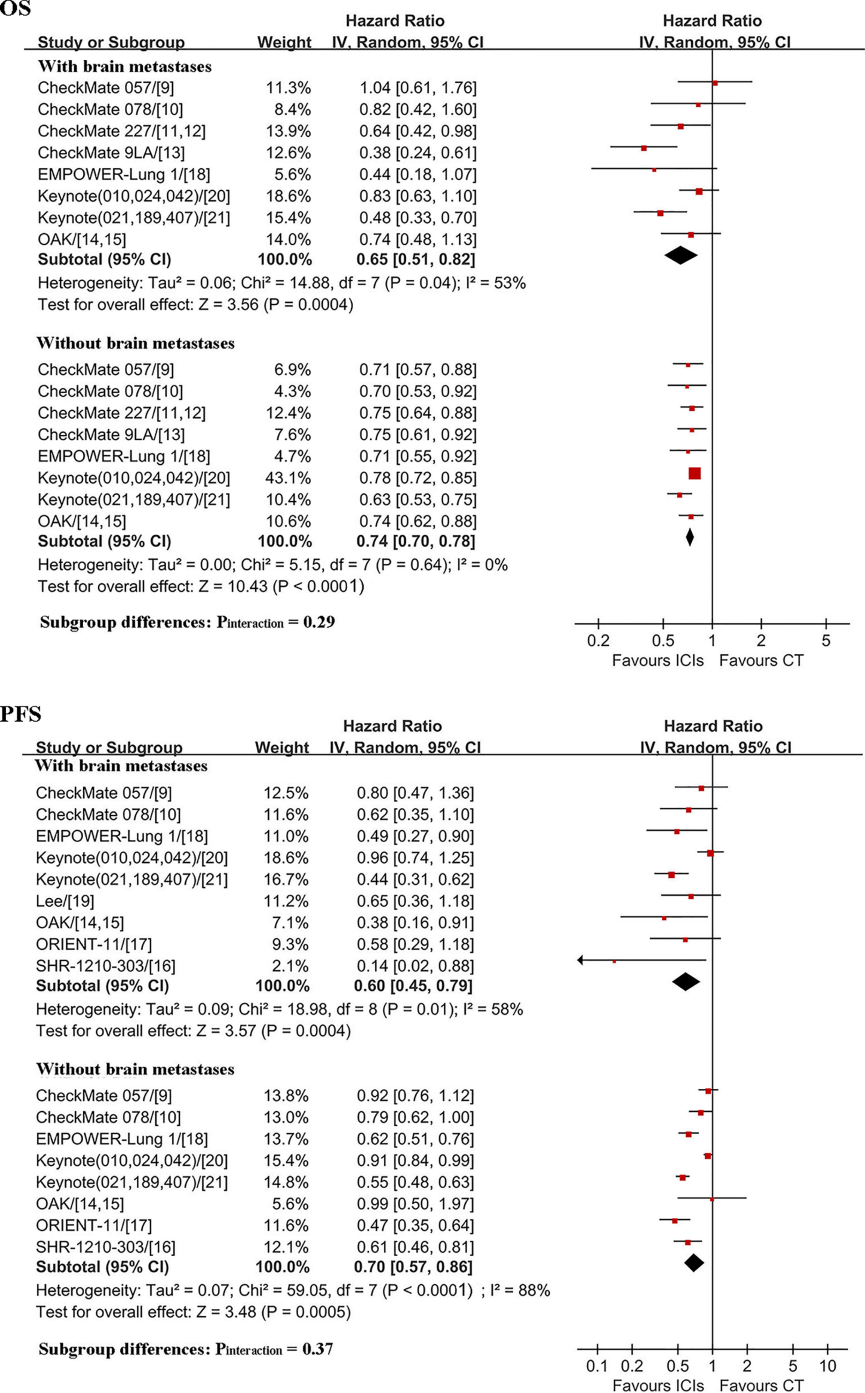
Comparison of ICIs efficacy between patients with and without brain metastases. OS, overall survival; PFS, progression-free survival; ICIs, immune checkpoint inhibitors; CT, chemotherapy; HR, hazard ratio; CI, confidence interval.

ICIs were associated with significantly longer OS and PFS than those in CT either in patients with (n = 1048; HR, 0.65; 95% CI, 0.51–0.82 and n = 961; HR, 0.60; 95% CI, 0.45–0.79) or without BMs (n = 7952; HR, 0.74; 95% CI, 0.70–0.78 and n = 7038; HR, 0.70; 95% CI, 0.57–0.86); no significant differences were observed in the pooled HRs for OS (P_interaction_ = 0.29) and PFS (P_interaction_ = 0.37) between the two patient populations (**Figure 2**). Heterogeneity was observed for OS (I^2^ = 53%, P = 0.04) and PFS (I^2^ = 58%, P = 0.01) in patients with BMs and for PFS in patients without BMs (I^2^ = 88%, P < 0.0001) (**Figure 2**).

### Subgroup Analyses

**Figure 3:**
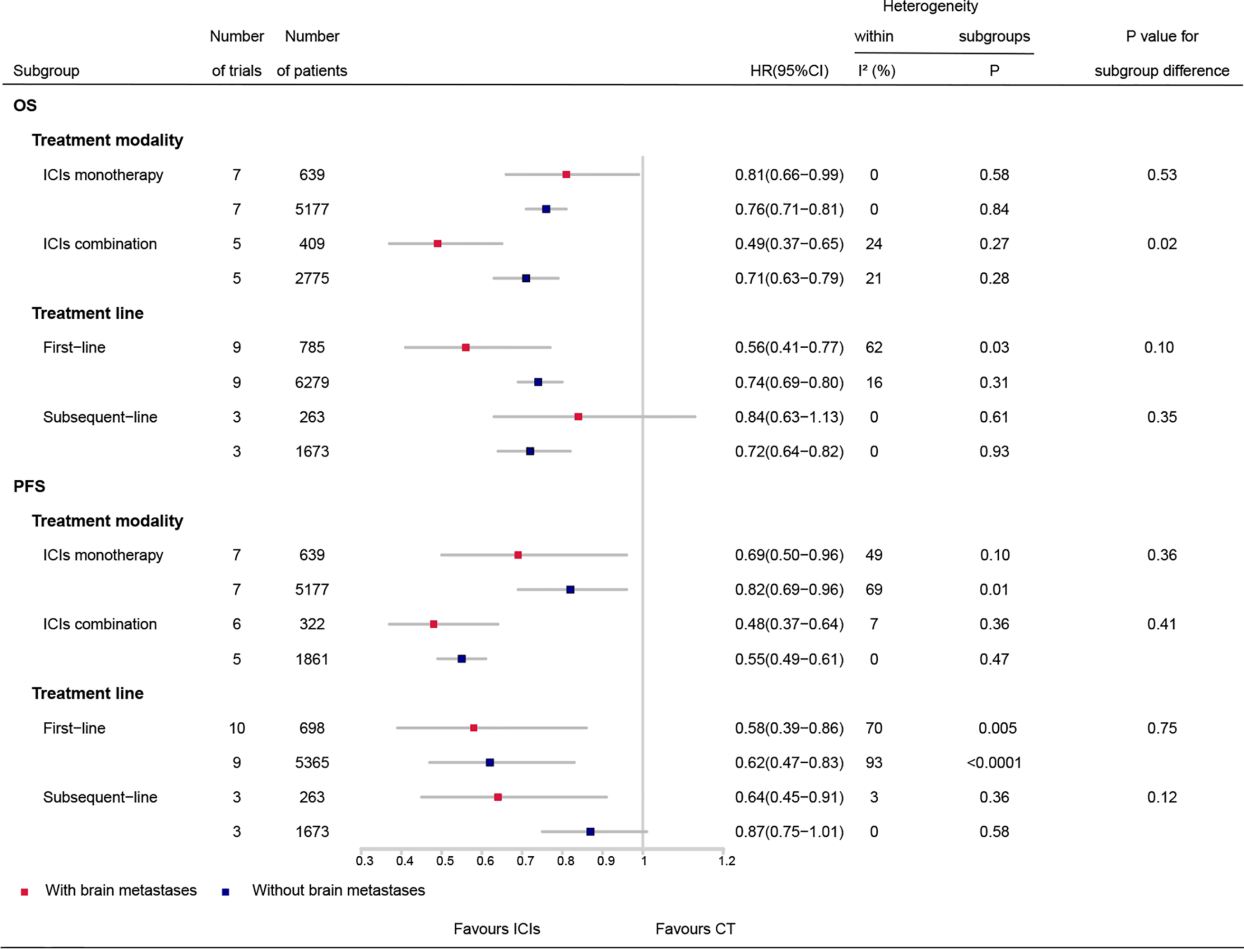
Comparison of ICIs efficacy between patients with and without brain metastases by subgroups. OS, overall survival; PFS, progression-free survival; ICIs, immune checkpoint inhibitors; CT, chemotherapy; HR, hazard ratio; CI, confidence interval.

Results of ICI efficacy in patients with and without BMs according to subgroups are shown in **Figure 3**. ICI monotherapy, ICI combination therapy, and first-line treatment with ICIs significantly improved OS and PFS compared with that in CT both for patients with and without BMs (with the HR and upper limit of the 95% CI smaller than 1 for each comparison). Subsequent-line treatment with ICIs was correlated with significant improvement in OS for patients without BMs (HR, 0.72; 95% CI, 0.64–0.82) but not for those with BMs (HR, 0.84; 95% CI, 0.63–1.13), whereas significant improvement in PFS was observed for patients with BMs (HR, 0.64; 95% CI, 0.45–0.91), but not for those without BMs (HR, 0.87; 95% CI, 0.75–1.01). As there were four subgroups either for OS or PFS, a *P-value* < 0.013 (0.05÷4) was considered to be statistically significant for interaction tests. As such, there was no significant difference in OS and PFS benefit between patients with and without BMs in each subgroup, including ICI combination therapy (OS: HR, 0.49 *vs.* 0.71; P_interaction_ = 0.02; PFS: HR, 0.48 *vs.* 0.55; P_interaction_ = 0.41), ICI monotherapy (OS: HR, 0.81 *vs.* 0.76; P_interaction_ = 0.53; PFS: HR, 0.69 *vs.* 0.82; P_interaction_ = 0.36), first-line treatment with ICIs (OS: HR, 0.56 *vs.* 0.74; P_interaction_ = 0.1; PFS: HR, 0.58 *vs.* 0.62; P_interaction_ = 0.75), and subsequent-line treatment with ICIs (OS: HR, 0.84 *vs.* 0.72; P_interaction_ = 0.35; PFS: HR, 0.64 *vs.* 0.87; P_interaction_ = 0.12). There was also no significant difference in OS benefit in subgroups of ICI monotherapy with PD-1 inhibitors, ICI monotherapy in patients with PD-L1 expression ≥1%, dual ICIs combination (**Supplementary File**: **Figure S3**).

**Figure 4:**
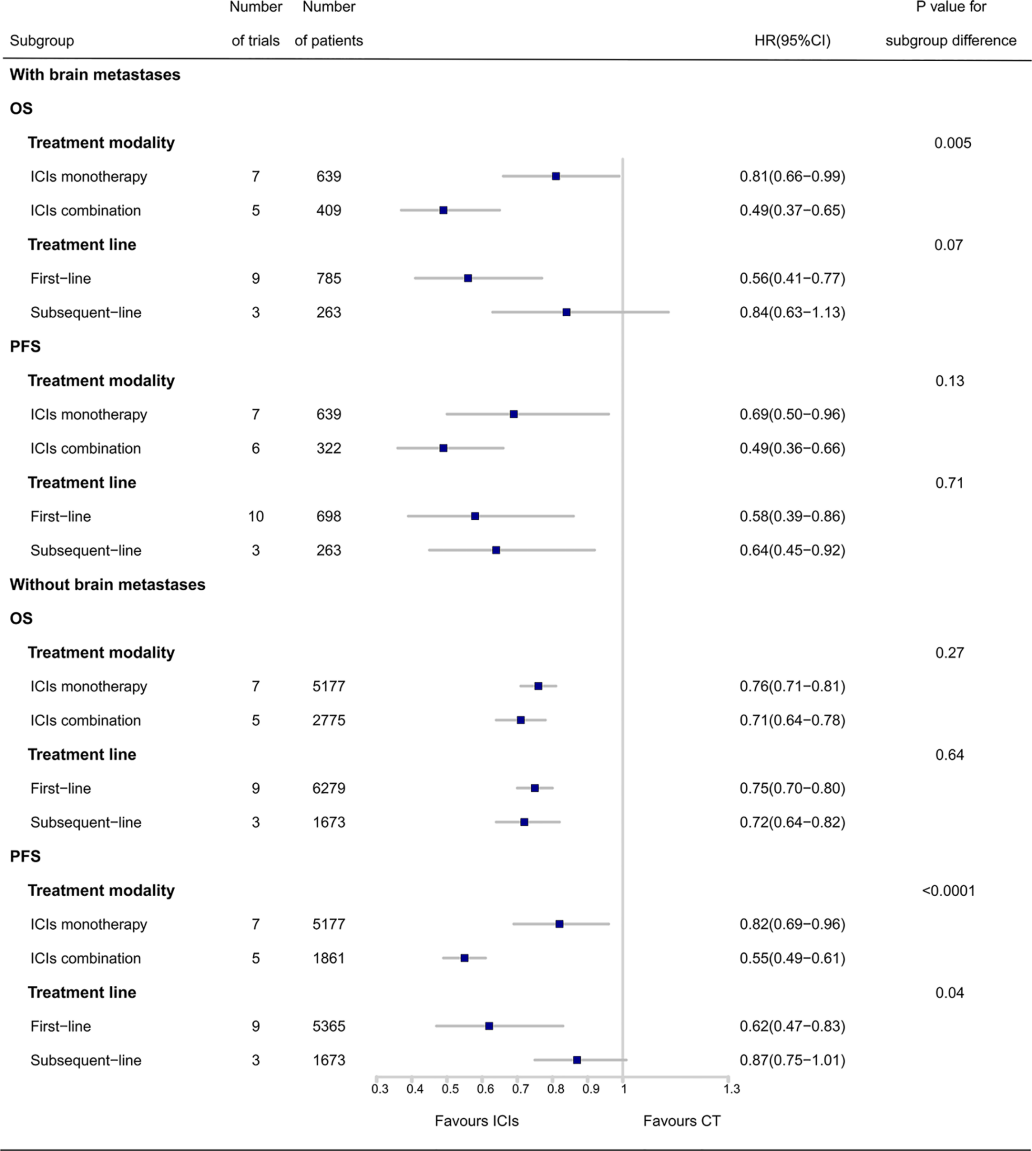
Subgroup analyses of ICIs efficacy in patients with and without brain metastases. OS, overall survival; PFS, progression-free survival; ICIs, immune checkpoint inhibitors; CT, chemotherapy; HR, hazard ratio; CI, confidence interval.

Subgroup analyses in patients with and without BMs are detailed in **Figure 4**. As there were two subgroups for OS or PFS in patients with or without BMs, a *P-value* < 0.025 (0.05÷2) was considered to be statistically significant for interaction tests. For patients with BMs, a greater OS benefit from ICI combination therapy than that from ICI monotherapy was observed (HR, 0.49 *vs.* 0.81; P_interaction_ = 0.005). No significant difference in OS benefit between first-line treatment with ICIs and subsequent-line treatment with ICIs was observed (HR, 0.56 *vs.* 0.84; P_interaction_ = 0.07). There were also no significant differences in PFS benefit between ICI combination therapy and ICI monotherapy (HR, 0.49 *vs.* 0.69; P_interaction_ = 0.13), and first-line treatment with ICIs and subsequent-line treatment with ICIs (HR, 0.58 *vs.* 0.64; P_interaction_ = 0.71).

For patients without BMs, no significant differences in OS benefit from ICIs were observed between ICI combination therapy and ICI monotherapy (HR, 0.71 *vs.* 0.76; P_interaction_ = 0.27), and first-line treatment with ICIs and subsequent-line treatment with ICIs (HR, 0.75 *vs.* 0.72; P_interaction_ = 0.64). However, ICI combination therapy achieved superior PFS compared with that in ICI monotherapy (HR, 0.55 *vs.* 0.82; P_interaction_ < 0.0001). There were no significant differences in PFS benefit between first-line and subsequent-line treatment with ICIs (HR, 0.62 *vs.* 0.87; P_interaction_ = 0.04).

### Sensitivity Analysis

Results of sensitivity analysis are shown in **Supplementary File**, **Figure S4**. When individual studies were removed one at a time from the analyses for OS and PFS, the corresponding pooled HRs were not markedly altered by any single study (HR lies between 0.61 and 0.70 for OS, and between 0.53 and 0.66 for PFS), indicating q relatively good stability of the presented results.

## Discussion

Currently, ICIs have been the standard first-line treatments for metastatic NSCLC lacking sensitizing EGFR or ALK or other druggable mutations. However, whether the presence of asymptomatic and/or treated can decrease the survival benefit from ICIs remains uncertain. This is a comprehensive meta-analysis focusing on the effect of asymptomatic and/or treated BMs on the efficacy of ICIs in metastatic NSCLC. This study included 15 RCTs involving 10358 patients (1,199 with and 9,159 without BMs). It showed that ICIs were associated with longer OS and PFS than that in CT either in patients with or without BMs; no significant differences in the pooled HRs for OS (HR, 0.65 *vs.* 0.60; P_interaction_ = 0.29) and PFS (HR, 0.74 *vs.* 0.70; P_interaction_ = 0.37) were observed, suggesting a comparable efficacy of ICIs for the two patient populations.

The exact mechanism of action of ICIs in the central nervous system (CNS) is yet to be determined; however, It is likely related to modified immune cell activity rather than direct action in the brain (27), and immune cell trafficking (28) and T-cell priming in the extracranial compartment could be essential for producing an effective immune response in the CNS (29). Moreover, lymphatic vessels in the dura mater were found to be potentially capable of allowing CNS antigen presentation in the peripheral lymph nodes (30), which might be another potential mechanism of action. Recently, several studies have reported a good activity of ICIs in CNS (31, 32). In a phase II trial, pembrolizumab resulted in a 33% objective CNS response rate in NSCLC patients with untreated BMs (31). An exploratory analysis of the phase III OAK study in patients with asymptomatic/treated BMs showed that new brain lesion-free probability at 24 months was 76.6% for atezolizumab and 0% for docetaxel (15). The additional intracranial activity of ICIs might be an explanation for our finding that patients with asymptomatic and/or treated BMs could obtain similar survival benefits from ICIs to patients without BMs.

The choice of monotherapy or combination therapy is an important factor that could affect the efficacy of ICIs in metastatic NSCLC. Current NCCN guidelines have recommended ICI monotherapy only for patients with high PD-L1 level, such as tumor proportion score (TPS) ≥50%, whereas ICIs in combination with CT is recommended, regardless of PD-L1 expression (2). However, PD-L1 expression of BMs sites can be different from primary lung tumors because of the distinct immune microenvironment of CNS (33). Whether the PD-L1 level of the primary tumor can work as a predictor of the efficacy of ICIs in patients with BMs remains uncertain. In a phase 2 trial of pembrolizumab in NSCLC or melanoma patients with untreated BMs, 29.7% of patients with PD-L1 expression ≥1% had a brain metastasis response, but no responses were observed in those with PD-L1 expression <1% or unevaluable (32). In a pooled analysis of KEYNOTE-010 and -024 and -042 trials (20), although pembrolizumab improved clinical outcomes compared with that in CT in PD-L1 positive patients (TPS ≥1%), no survival benefits were observed for those with asymptomatic/treated BMs at baseline. Our study did not assess the correlation between PD-L1 expression and the efficacy of ICIs due to few studies reporting PD-L1 status for patients with BMs. In subgroup analyses of treatment modality, both ICI monotherapy and combination therapy achieved significantly longer OS and PFS compared with that in CT in patients with BMs, whereas a greater OS benefit from combination therapy was observed (HR, 0.49 *vs.* 0.81; P_interaction_ = 0.005). Unexpectedly, we also found that patients with BMs could obtain more OS benefits from ICI combination therapy than that in patients without BMs. Despite our inability to provide a satisfactory explanation for this result, ICI combination therapy was likely to be the optimal choice for patients with asymptomatic and/or treated BMs based on the results above. Nevertheless, these findings need to be confirmed in large phase III trials.

Besides the first-line treatment with ICIs, several trials investigated the efficacy of ICI monotherapy as a subsequent-line treatment in NSCLC patients with treated BMs. Two phase III trials demonstrated that nivolumab achieved superior OS compared with that in docetaxel in previously treated advanced NSCLC, but the OS benefit was not observed in the subgroup of patients with treated, stable BMs at baseline (9, 10). However, in the exploratory analyses of the phase III OAK study (15), subsequent-line treatment with atezolizumab gained a trend OS benefit compared with that in docetaxel (HR, 0.74; 95% CI, 0.49–1.13) in patients with a history of asymptomatic or treated BMs. In our meta-analysis, subsequent-line treatment with ICIs significantly improved PFS compared with that in CT but failed to show a significant OS benefit in patients with asymptomatic and/or treated BMs. Whether subsequent-line treatment with combinations of immunotherapy, such as dual ICI combination or ICIs in combination with antiangiogenic agents, could be more effective in this patient population requires further investigation.

The selection of PD-1 or PD-L1 inhibitors might be another factor that influences the efficacy of ICIs. Results of a more recent meta-analysis showed that anti-PD-1 achieved superior OS and PFS compared with those in anti-PD-L1 in cancer patients (34). However, whether there is a difference in intracranial activity between the two ICI classes in NSCLC patients with BMs remains unclear. Since there was only one included trial providing information on PD-L1 inhibitors, we did not compare the efficacy of PD-1 with PD-L1 inhibitors for this patient population.

In fact, our meta-analysis included two types of BMs: previously treated or untreated asymptomatic BMs, and previously treated and stable symptomatic BMs. For patients with asymptomatic BMs, whether upfront brain irradiation before the start of ICI therapy is needed remains unclear because of the paucity of clinical trials assessing this. In a recent retrospective study on PD-L1, in ≥ 50% of advanced NSCLC patients treated with first-line pembrolizumab (35), a high intracranial response rate (iRR) of 67.5% was observed in patients with BMs. Of note, 80.0% (32/40) of the patients with BMs received brain irradiation prior to treatment with pembrolizumab, which might contribute to the high iRR. However, an iRR of 75% (6/8) was still observed in those without prior brain irradiation because their BMs were asymptomatic. In addition, Wakuda et al. also retrospectively reviewed NSCLC patients with PD-L1 ≥ 50% receiving first-line pembrolizumab (36). In their study, the BM group was divided into patients who previously received radiation for BMs before pembrolizumab (BM-T group) and those with no prior radiation for BMs (BM-not T group); and there were 53% (7/13) and 100% (10/10) patients with asymptomatic BMs in BM-T and BM-not T groups, respectively. They found that there was no significant difference in treatment efficacy between the BM-T and BM-not T groups. These findings suggest that upfront brain irradiation before first-line treatment with pembrolizumab may be spared for PD-L1 ≥ 50% NSCLC patients with asymptomatic BMs, whereas this strategy needs to be confirmed in phase 3 trials. Meanwhile, there is also a need to assess the value of brain irradiation prior to ICI therapy for asymptomatic BMs with low/negative PD-L1 expression in further trials.

A recently published pooled analysis of metastatic NSCLC patients (including 255 patients with BMs) from seven European centers investigated predictors of the efficacy of ICIs in patients with BMs (37). Active BMs (defined as patients with previously untreated BMs or patients with brain involvement that have progressed after previous local therapy), lower disease-specific Graded Prognostic Assessment (ds-GPA) score, and use of corticosteroids at the start of ICIs treatment were associated with poorer OS in multivariate analysis in the BMs subgroup (37). The patients with active BMs had brain PD significantly more often than that in patients with stable BMs (54.2% *vs.* 30%, p <0.001). Among patients with active BMs, PD-L1 expression ≥1% was associated with a higher intracranial RR: 35.7% *vs.* 11.1% in PD-L1-negative patients (37). These results may help clinicians in the decision of whether to administer ICIs to a patient with NSCLC who has BMs. Nevertheless, given the retrospective nature of this analysis, the findings need to be confirmed by more robust clinical trials.

Currently, there are still insufficient unified criteria to assess the intracranial response in patients with BMs undergoing ICIs. Conventional methods, such as RECIST and WHO, evaluate tumor response only depending on the tumor shrinkage within a few weeks of initiating treatment (38). However, immunotherapy might demonstrate a delay in response, transient enlargement followed by tumor shrinkage, stable size, or the appearance of new lesions (39). Unlike the WHO and RECIST criteria, the modified immune-related Response Criteria (irRC) and immune-Related RECIST (irRECIST) criteria take the delayed response and new measurable lesions into account (39). Nevertheless, the two new criteria are mainly used for solid tumors of the whole body. RANO-BM was developed for assessing the therapeutic response of brain metastasis only. Intracranial response evaluation is based on a combination of tumor measurements, clinical status, and corticosteroid use (40). The use of immunotherapy in metastatic brain tumors leads to modifications in the RANO-BM criteria for these patients (iRANO-BM) (41). iRANO-BM is now thought to be a representative assessment criterion considering intracranial pseudoprogression after immunotherapy (41, 42).

Several previous meta-analyses (43–45) of metastatic NSCLC also investigated the efficacy of ICIs in patients with asymptomatic and/or treated BMs in subgroup analyses. However, a maximum of three trials was included in those studies for assessing this subgroup of patients, which would result in poor accuracy. Our meta-analysis specifically addressed this subject and included 11 additional RCTs (including six more recent phase 3 trials presented at the meeting of the ESMO/ASCO/WCLC in 2019 and 2020). Moreover, we performed subgroup analyses of ICI monotherapy, ICI combination therapy, first-line treatment with ICIs, and subsequent-line treatment with ICIs and compared the efficacy of ICIs between patients with and without BMs. The present meta-analysis would be more comprehensive in assessing the effect of asymptomatic and/or treated BMs on the efficacy of ICIs in metastatic NSCLC.

Nevertheless, our meta-analysis has several limitations. First, despite all included studies being RCTs and most of them being phase III trials, all data of patients with and without BMs were extracted from subgroup analyses of these RCTs, which might result in a potential imbalance in baseline characteristics between the two sets of patients. Second, some RCTs, such as IMpower series studies (46–50) and CheckMate 017 (51) and 026 (52), were excluded from our study because of the non-reporting of survival information of patients with BMs. This might result in a selection bias to some extent. Third, heterogeneity was observed for OS and PFS in patients with BMs, and for PFS in patients without BMs. Results of subgroup analyses suggested that treatment line and treatment modality may be two potential sources of heterogeneity. In addition, chemotherapy regimens were inconsistent among studies, which might also lead to heterogeneity. Finally, this study only assessed patients with asymptomatic and/or treated BMs; therefore, the conclusion should be interpreted with caution for patients with symptomatic, untreated brain disease.

In conclusion, there was no compelling statistical evidence that the efficacy of ICIs in metastatic NSCLC was modified by the presence of asymptomatic and/or treated BMs. Patients with BMs were likely to obtain more OS benefits from ICI combination therapy than that from monotherapy. Further RCTs specifically on this subject are needed to confirm these findings.

## Data Availability Statement

The original contributions presented in the study are included in the article/**Supplementary Material**. Further inquiries can be directed to the corresponding author.

## Author Contributions

Conception and design: JD. Collection and assembly of data: SL and HZ. Data analysis and interpretation: all authors. All authors contributed to the article and approved the submitted version.

## Conflict of Interest

The authors declare that the research was conducted in the absence of any commercial or financial relationships that could be construed as a potential conflict of interest.
